# Robust Grape Detector Based on SVMs and HOG Features

**DOI:** 10.1155/2017/3478602

**Published:** 2017-05-18

**Authors:** Pavel Škrabánek, Petr Doležel

**Affiliations:** Department of Process Control, University of Pardubice, Pardubice, Czech Republic

## Abstract

Detection of grapes in real-life images is a serious task solved by researchers dealing with precision viticulture. In the case of white wine varieties, grape detectors based on SVMs classifiers, in combination with a HOG descriptor, have proven to be very efficient. Simplified versions of the detectors seem to be the best solution for practical applications. They offer the best known performance versus time-complexity ratio. As our research showed, a conversion of RGB images to grayscale format, which is implemented at an image preprocessing level, is ideal means for further improvement of performance of the detectors. In order to enhance the ratio, we explored relevance of the conversion in a context of a detector potential sensitivity to a rotation of berries. For this purpose, we proposed a modification of the conversion, and we designed an appropriate method for a tuning of such modified detectors. To evaluate the effect of the new parameter space on their performance, we developed a specialized visualization method. In order to provide accurate results, we formed new datasets for both tuning and evaluation of the detectors. Our effort resulted in a robust grape detector which is less sensitive to image distortion.

## 1. Introduction

Detection of grapes in real-life images is a serious task solved by many researchers dealing with precision viticulture [1]. Grape detectors are employed in various applications, for example, in autonomous vineyard sprayers and harvesters or in the process of yield estimation [2–5]. Various types of image processing, feature extraction, and classification algorithms can be employed when detecting berries or bunches of grapes in RGB images.

A bunch detector designed by Reis et al. [6] employs colour mapping, morphological dilation, and stem detection. It showed correct white wine bunch classification at 90.53% and red wine at 97.14%, demonstrating an increase in complexity of detection depending on grape colour. Thus, let us focus only on solutions aimed at white variety detection.

A detector introduced by Berenstein et al. [2] was based on the decision tree algorithm and was applicable for both the bunch and the berry detection. Its detection rate of bunches was 90.45%, and the detection rate of single grapes was 90.10%. An exceptional single grape detector was developed by Nuske et al. [3]. It showed overall precision at 98.00% but recall was lower at 63.70%. Their detector utilized radial symmetry transformation, Gabor filters, and a *k*-nearest neighbours classifier.

A comparable method [7], aimed at detection of white wine varieties, considered support vector machines (SVMs) classifiers in combination with histograms of oriented gradients (HOG). Specifically, its average accuracy by a 10-fold cross-validation (CV) was 98.23% for a linear kernel and 98.96% for a radial basis function (RBF) kernel. Similar results were achieved in their evaluation on real-life images [8].

While excellent, these detectors are computationally intensive to an extent that makes them impractical in viticulture applications. Modification of an image preprocessing (IP), where an input RGB image is first converted to a grayscale format and then adjusted with a linear contrast normalization, can be used to reduce the time complexity of the original solutions [9]. The impact of the normalization step was shown to be negligible in the grape detectors [8, 9], but appropriateness of the grayscale conversion remains to be tested.

The evaluation of the detectors requires sets of labelled object images. These sets might significantly influence evaluation results. Škrabánek et al. [7, 9] discovered inconsistency in the performance of the detectors that depended on the evaluation set. Although the detectors have a high score in the 10-fold CV and an evaluation on real-life images, they do not achieve such results on test sets [7]. The test sets contained distorted positive samples, where 75% of the positive samples in each test set were artificial samples created by a rotation of original samples. The detectors were tested on two categories of test sets. The poorer grape detection results were present for both categories. For example, the average accuracy of the original detector with the linear kernel was as low as 87.85% in a test set [7].

Here, we address both the relevance of the grayscale conversion and a potential sensitivity of the detectors to the image rotation. We test a modification of the conversion and propose a parameter tuning method, designed for such modified grape detectors. To evaluate the effect of the new parameter space on their performance, we developed a specialized method for a visualization of the evaluation results. We applied the modified conversion and the tuning method into grape detectors developed by Škrabánek and Majerík [9], and we evaluated performance of these modified versions. In order to provide accurate results, we formed new datasets for both tuning and evaluation of the detectors. Our improvements resulted in a robust grape detector which is less sensitive to image distortion.

## 2. Materials and Methods

### 2.1. Original Work on Grape Detectors

The presented study evaluates and modifies previous research published in [7, 9]. Herein, we provide a brief summary of the published grapes detectors and the test sets.

#### 2.1.1. Objective of the Detectors

The grape detectors were aimed at the recognition of grapes of white varieties in object images. RGB object images *I* of dimensions 40 × 40 px (pixel) were considered in [7–9]. The object images were square viewports of source images of a resolution 1936 × 1288 px, 24 bit.

The detectors distinguished between two classes *y*: “berry” and “not berry.” The class “berry” was called “positive” and the class “not berry” was called “negative.” Object images belonging to the class “positive” contained berries of the circle shape of a diameter ranging between 30 and 40 px. Moreover, the middle of the berries was required to be placed in the middle of the object images with a tolerance ±1 px. Object images, which did not satisfy this condition, belonged to the class “negative.”

#### 2.1.2. Structure of the Detectors

**Figure 1:**
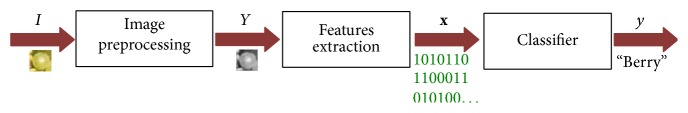
Vision pipeline of the grape detectors.

In computer vision, detection of objects in images usually consists of four successive steps. The first step is acquiring an object image *I* from a large real-life image; the second step is the IP resulting in a modified image *Y*; the third one is extraction of features; and the final step is classification of the object image using a feature vector **x**. However, the grape detectors introduced in [7, 9] consist specifically of three parts only: from the IP, a features descriptor, and a classifier. Although the detectors differ in structure of the IP, used feature descriptors, and settings of classifiers, they have the same arrangement of a vision pipeline (Figure 1). Parts of the detectors are described in the context of our previous works in further details.

*Image Preprocessing*. IPs of the original detectors, labelled as O [7], consist of two steps: the conversion of input RGB images to grayscale format followed by a linear contrast normalization on the range [0,1]. Simplified versions, which were introduced in [9], skip either the contrast normalization (simplified version 1 or S_1_) or both operations (simplified version 2 or S_2_). In O and S_2_, the conversion is carried out according to ITU-R recommendation BT.601 [10]. This conversion belongs to techniques based on weighted means of all three colour channels [11]. It means that the conversion of the input RGB image *I* from the RGB model to the grayscale format is realized by eliminating the hue and saturation information, while retaining the luminance. 

*Features Extraction*. Two types of features, a vector of normalized pixel intensities and HOG, were considered in [7]; however, only the HOG features have proven to be convenient for the detection of grapes of white varieties [7–9]. Thus, only the detectors based on the HOG features will be considered further. It means that the feature vectors **x** are extracted from *Y* using the HOG descriptor [12]. A standard setting of the descriptor has demonstrated to be sufficient. Specifically, a linear gradient voting into 9 bins in 0°–180°; cells of size 6 × 6 px; blocks of 2 × 2 cells; and 1 overlapping cell between adjacent blocks in both directions were used in [7–9]. 

*Classifier*. The aim of classifiers in the grape detectors is a judging the classes *y* of the object images *I* using feature vectors **x**. SVM classifiers [13] with the linear and the RBF kernel functions were used in [7, 9]. Regardless of the used kernel function, the performance of a SVM classifier is influenced by a regularization constant *C*. Performance of a classifier with the RBF kernel is further influenced by a kernel width *σ*. A grid search algorithm [14] combined with the 10-fold CV was used to find settings of these parameters giving the maximal recognition accuracy [7].

#### 2.1.3. Training and Evaluation of the Detectors

Five training sets were introduced in [7]. The *i*th training set was denoted as T-*i*, where *i* ∈ *X*, and *X* = {1,2,…, 5}. Each training set consisted of 288 unique “positive” and 288 unique “negative” samples. The training set T-3 was used for the training of the detectors [7–9].

Three kinds of evaluation methods were considered by the evaluation of the original detectors O [7, 8]: the 10-fold CV, an evaluation on test sets, and an evaluation on cut-outs of one vineyard photo. The simplified detectors S_1_ and S_2_ were evaluated using the test sets [9] and the cut-outs [8]. In all cases, three performance measures *m* were used for the evaluation:

(1a) accuracy=TP+TNTP+FN+TN+FP·100,

(1b) precision=TPTP+FP,

(1c) recall=TPTP+FN,

where |TP| is the number of correctly classified “positive” samples, |FN| is the number of misclassified “positive” samples, |FP| is the number of misclassified “negative” samples, and |TN| is the number of correctly classified “negative” samples [15]. Naturally, for the cut-outs, unbiased variants of the measures have been used [8].

While just the training sets were needed for the 10-fold CV, appropriate datasets must be created for the remaining two evaluation methods. Creation of these datasets was sufficiently described in [7]; however, datasets used for the evaluation on test sets should be detailed here. For this purpose, two types of datasets were created: an* environment type* E and a* grape type* G. Five sets of each type were formed [7]. The *i*th test set of the type E was denoted as E-*i* and the *i*th test set of the type G as G-*i*, where *i* ∈ *X*.

Each test set consisted of 200 “positive” and 200 “negative” samples. The sets were based on one vineyard row photo which was not used for creation of the training sets. To form a single test set, 50 unique “positive” and 200 unique “negative” samples were used. Each test set was further extended by artificial “positive” samples [16]. The “positive” samples were created by turning of the images through an angle *φ*, where *φ* ∈ {0, *π*/2, *π*, 3*π*/2}.

**Figure 2:**
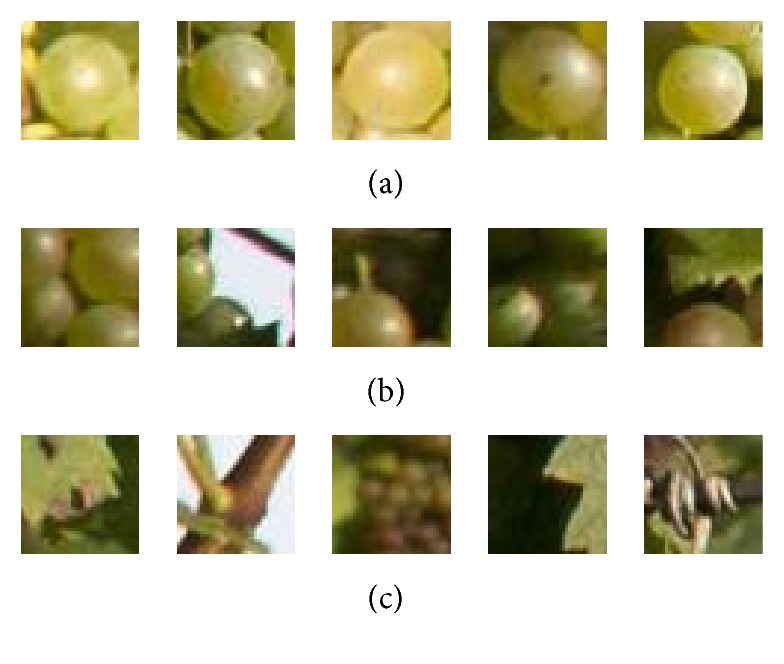
Examples of object images of the class: (a) “positive,” (b) “negative,” grape type, and (c) “negative,” environment type.

The difference between these two types of test sets consisted in the selection of the “negative” samples. The “negative” samples in G were composed solely of incomplete grape berries of a diameter ranging between 30 and 40 px, while the “negative” samples in E were based on the environment only, and they did not capture even the smallest piece of the targeted berry. Examples of “positive” samples as well as of both types of “negative” samples are shown in Figure 2.

### 2.2. Robust Grape Detector

While the original, as well as the simplified, grape detectors showed excellent performance by the 10-fold CV [7] and by the evaluation on the cut-outs [8], considerably worse results were obtained by their evaluation on the test sets [7, 9]. For example, the original version of the detector with the linear kernel reached the following average score by the 10-fold CV: precision = 0.980, accuracy = 98.23%, and recall = 0.987. The following average results were obtained for the same detector on the test sets of the type E: precision = 0.977, accuracy = 87.85%, and recall = 0.776. Similar results were obtained on both types of test sets for all versions of detectors.

The drop in accuracy and recall might be caused by different reasons. Generally, the most common source of a discrepancy in evaluation results is an inadequacy of datasets used by the evaluation. In the case of the grape detectors, a distortion of positive samples caused by the rotation might be considered as well. Indeed, the HOG features are not rotation invariant [12]. Since a potential sensitivity of the detectors on the distortion caused by the rotation of images is undesirable, we started a research aimed on enhancement of the detector's robustness.

Two principally different means might help to improve the detector's performance in the presence of a distortion: either a more appropriate training set could be used for the training, or a modification in the vision pipeline could be done. In our research, we focused on the modification of the pipeline (Section 2.2.1). Unfortunately, three additional tenable parameters were introduced by the presented modification, which considerably complicated the search for the optimal setting of the modified detector. In order to face this disadvantage, we developed a specialized tuning methodology (Section 2.2.3). The methodology was based on a visualization method which we designed for this purpose (Section 2.2.2).

#### 2.2.1. Modification of the Detector

Although the selection of a good feature vector is widely recognized to be fundamental when designing image recognition systems, the IP may also significantly influence the performance of a final solution. Thus, an intelligent use of the IP can provide benefits and solve problems that ultimately lead to better local and global feature detection [17].

IPs of the original grape detectors consisted of two operations: the conversion of RGB images to the grayscale format and the linear contrast normalization. Our later research has positively shown that the skipping of the contrast normalization does not influence the performance of the detectors at all [9]. Nonetheless, the relevance of the conversion in the detectors was not disproved.

Importance of the conversion of RGB images to the grayscale format, in connection with the image recognition systems, was studied, for example, by Kanan and Cottrell [11]. They have shown that a method used by the conversion of colour images to the grayscale format may significantly influence performance of the image recognition system, even when using robust descriptors. Further, they have pointed out the fact that different tasks involve different conversion methods. For object recognition tasks, they recommend techniques based on weighted means of the red, green, and blue image channels.

Considering their outcomes, we proposed to use a general formula for the conversion based on the weighted means. The main motivation for its use is the fact that this formula allows better control of the conversion, which consequently may allow us to improve the performance of the detectors. The general formula can be written as

(2) $$\begin{array}{c} I^{*}=w_{R}I_{R}+w_{G}I_{G}+w_{B}I_{B}, \end{array}$$

where *I*_R_, *I*_G_, and *I*_B_ are intensity images of the red, green, and blue components of the RGB image *I*; and *w*_R_, *w*_G_, and *w*_B_ are weights of the colour components in the resulting grayscale image *I*^*∗*^. It holds that *w*_R_, *w*_G_, *w*_B_ ∈ [0,1] and *w*_R_ + *w*_G_ + *w*_B_ = 1. Since conversion (2) is the single operation performed within IPs of the modified detectors, it holds that *Y* = *I*^*∗*^.

We modified the simplified version S_1_ of the grape detector using the general formula (2); that is, the standard conversion was replaced by this formula. The rest of the vision pipeline was not changed; that is, the new detector consisted of the HOG descriptor and the SVM classifier. We named the new detector as a* robust grape detector* or simply R.

Considering the essence of the proposed modification, it is apparent that the modification does not increase the computational complexity of the detector. Inherently, it may not cause a worsening of its performance. Indeed, the setting of the weighting coefficients according to the ITU-R recommendation BT.601 is just one of an infinite number of possible settings.

#### 2.2.2. Performance Visualization for Better Understanding of Tuning Parameter Relevance

While searching for the optimal setting of an image recognition system, an appropriate variant of the grid search algorithm is typically used. For just one or two tuneable parameters, meaningfulness of evaluation results can be assessed on the basis of the raw data directly by a specialist. However, the assessment is becoming challenging when increasing the number of tuneable parameters.

Considering the parameters of the HOG descriptor to be nontuneable, the original and the simplified versions of the detectors have only one or two tuneable parameters (depending on the used kernel function). The fixation of the HOG descriptor setting has its origin in practical reasons. The setting of the HOG descriptor, which was summarized in Section 2.1.2, is appropriate in terms of exactitude versus computational complexity. Thus, this fixed setting is used for the robust detectors as well.

The original tuneable parameters, *C* and *σ*, were extended by three additional parameters, *w*_R_, *w*_G_, and *w*_B_, in the case of the robust grape detectors. These parameters were introduced by formula (2). Thus, the tuning of a robust grape detector setting using a grid search algorithm results in a large amount of data. An analysis of such quantities of data in the raw numeric form is really not convenient for humans. In order to facilitate the analysis, we suggested a ternary diagram for a visualization of the data. Specifically, we used the diagram to show a prospective influence of the weighting coefficients, *w*_R_, *w*_G_, and *w*_B_, on the performance of an overall solution for a fixed setting of the remaining tuneable parameters.

The ternary diagram is a graph which consists of an equilateral triangle in which a given plotted point represents the relative proportions (*a*, *b*, *c*) of three end-members (*A*, *B*, and *C*), usually expressed as percentages (do not confuse the end-member *C* with the regularization constant *C*). Moreover, the sum of the relative proportions is equal to a given value; for example, for percentages, it holds that *a* + *b* + *c* = 100%. The axis related to the member *A* is the left arm of the triangle. The relative proportion of *A*, *a*, is plotted on the axis where *a* increases downwards. The same principle is used for the remaining two axes where the bottom axis is related to component *B* and the right one to component *C*. The relative proportion of *B* increases in the right direction and the relative proportion of *C* upwards. A dependent variable can be represented in different ways, for example, using contour plot or shaded surface. For more information, see [18].

In our case, the three end-members, *A*, *B*, and *C*, are the intensity images of the colours, *I*_R_, *I*_G_, and *I*_B_, respectively. The relative proportions, *a*, *b*, and *c*, are the weights, *w*_R_, *w*_G_, and *w*_B_, where *w*_R_, *w*_G_, *w*_B_ ∈ [0,1]. Moreover, the weights are bounded by the condition *w*_R_ + *w*_G_ + *w*_B_ = 1. The ternary diagram is aimed to be used as a supporting tool by the evaluation of the grape detectors. Thus, a performance measure *m* is the dependent variable to be displayed. Considering our previous experience, we suggested to use the shaded surface for the visualization of the results. This type of diagram gives the better idea about the influence of the weights on the performance measure.

Let us denote a setting of the weighting coefficients **w** as an ordered triple of the weights *w*; that is, **w** = (*w*_R_, *w*_G_, *w*_B_). Further, let us form a finite set *W* of settings **w** for the purpose of the graph construction, and let us call the set* grid*. The grid should uniformly cover the surface bounded by the triangle. It means that a step Δ*w*, of a fixed size, has to be used by forming the grid. Let us express the step as Δ*w* = 1/*p*, where *p* > 0, *p* ∈ *ℕ*. Thus, the weighting coefficients *w* can take any value from {0, Δ*w*, 2Δ*w*,…, 1}; however, a combination of the coefficients in (*w*_R_, *w*_G_, *w*_B_) is bounded by the condition *w*_R_ + *w*_G_ + *w*_B_ = 1. It means that the grid *W* is a set of all admissible settings **w**.

**Figure 3:**
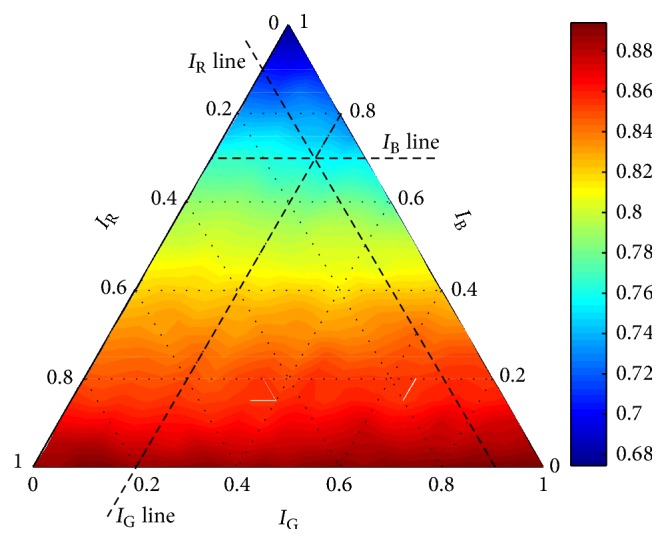
Demonstration of working with the ternary diagram.

In order to construct the ternary diagram, evaluation of a detector using a measure *m* has to be achieved for ∀**w** ∈ *W*. Naturally, the classifier has to be trained for each of these settings separately. Settings of the classifier (*C* for linear kernel and *C*, *σ* for RBF kernel) must not be changed within the training-evaluation process. Once the training-evaluation process is performed for all the admissible settings **w**, construction of one diagram can be executed, where the diagram shows dependence of the used performance measure *m* on the weights *w*_R_, *w*_G_, and *w*_B_ for one particular setting of *C* or *C*, *σ*. An example of the diagram is shown in Figure 3 where the recall of a grape detector is displayed.

Figure 3 can be used also for explanation of how to work with the diagram. Let us suppose that the recall for *w*_R_ = 0.1, *w*_G_ = 0.2, and *w*_B_ = 0.7 is required to be determined. The reading of the recall value can be done using auxiliary lines which are plotted using dashed lines in Figure 3. Each line is parallel with one of the sides of the triangle. The lines are named with respect to referred components; that is, “*I*_R_ line” is related to the intensity images of the red colour *I*_R_, “*I*_G_ line” to the intensity images of the green colour *I*_G_, and “*I*_B_ line” to the intensity images of the blue colour *I*_B_.

Positions of the lines are given by the weights *w*. In this example, “*I*_R_ line” passes the left axis at the point 0.1, “*I*_G_ line” passes the bottom axis at the point 0.2, and “*I*_B_ line” passes the right axis at the point 0.7. The intersection of the lines positively determines the recall. The numeric value can be estimated using the colour bar. In this example, the recall is approximately 0.78. It might be noted here that only two auxiliary lines are necessary for reading of the dependent variable. Indeed, the proportion of a third component is always positively determined by the constraint of their sum, in this case by *w*_R_ + *w*_G_ + *w*_B_ = 1.

#### 2.2.3. Tuning Methodology

The goal of our research was the development of a grape detector which would be invariant to the distortion caused by the rotation. One of the key steps within the development process is finding the setting of all tuneable parameters giving the best performance according to a criterion. The search for the optimal setting is usually executed using a variant of the grid search algorithm [14].

The grid search algorithms ensure a systematic evaluation of an image recognition system performance. Usually, the grid search is combined with the CV. In such a case, the evaluated system is trained on a part of a dataset and evaluated on the rest of the dataset. The training is carried out for various settings of all tuneable parameters. The settings might be assigned according to a rule or directly defined by an expert. The setting giving the best score is considered to be optimal. In order to obtain more accurate results, the search can be performed repeatedly. The search is then carried out with a finer resolution in a scaled down area. An area promising best results is selected for the finer search.

In order to develop a methodology aimed at tuning of the robust detectors, we adopted the basic principles of the grid search algorithms. Specifically, the training-evaluation process is carried out for various settings of all tuneable parameters within the search process. However, our methodology requires involvement of a computer vision specialist. Further, the evaluation of the image recognition system should be performed on a dataset affected by the target distortion. For that reason, the commonly used CV was replaced by the evaluation of the system on a specialized dataset.

In order to ensure flexibility of the method, we prepared the methodology for a multiple criteria usage. The proposed methodology allows combination of several performance measures when searching for the optimal setting. In the case of the multiple criteria, a priority of the performance measures has to be determined in advance. For the detector with the RBF kernel, the methodology consists of following steps:

(1) Select performance measures and give them priorities. In such a way, a finite set *M* of measures *m* is created where each measure is paired with a priority.
(2) Define admissible settings of all tuneable parameters *X*_*C*_, *X*_*σ*_, and *W*, where *X*_*C*_ is a finite set of all admissible settings of *C*, and *X*_*σ*_ is a finite set of all admissible settings of *σ*. Such a way, a parameter space Θ of features *θ* is formed, where *θ* ∈ Θ, and *θ* = (*C*, *σ*, **w**).
(3) Perform the training-evaluation process ∀*θ* ∈ Θ using ∀*m* ∈ *M*.
(4) Display the obtained results using the diagram. Such a way, |*M*| × |*X*_*C*_| × |*X*_*σ*_| graphs are obtained, where |•| denotes a cardinality of a set.
(5) Manually evaluate the obtained results using the graphs. Identify combinations of *C* and *σ* leading to senseless results. Eliminate all settings *θ* containing the offending combinations of *C* and *σ* from the further processing; that is, a new parameter space $\hat{\Theta }$ is formed where $\hat{\Theta }\subset \Theta$.
(6) For each performance measure *m* ∈ *M*, find the setting *θ*_*m*_^*∗*^ giving the best score according to
  (3) $$\begin{array}{c} \theta_{m}^{*}=\underset{\forall \theta \in \hat{\Theta }}{\arg\max\;}m\left(\;\theta \;\right). \end{array}$$
(7) Determine a globally optimal setting *θ*^*∗*^ on the basis of all *θ*_*m*_^*∗*^. Within this step, the priority of the measures must be taken into account; however, the functional dependence shown in the appropriate graphs must be considered as well.

This methodology can be also applied on the detector with the linear kernel. Naturally, the variable *σ* should be ignored in this case.

### 2.3. Design of Evaluation Experiments

In the experimental part, we evaluated relevance of the grayscale conversion and the potential sensitivity of the detectors to the image rotation. For this purpose, new statistically relevant datasets were created.

#### 2.3.1. Assessment of Conversion Importance

The assessment of the conversion relevance was one of the main goals of the presented work. Two versions of the conversion were considered in the grape detectors. While the grape detectors S_1_ employ the standard conversion according to the ITU-R recommendation BT.601, the robust detectors R are based on the generalized conversion (2). The detectors S_2_ do not perform any conversion within the IP.

Performances of all three variants of the detectors have to be confronted concerning the assessment of the conversion relevance. In order to keep comparability of the results, the detectors should be tuned the same way. Thus, when tuning S_1_ and S_2_, the methodology presented in Section 2.2.3 should be used in appropriately modified form. It means that steps  (4) and (5) have to be left out when searching for their settings.

#### 2.3.2. Dataset for Tuning

The proposed tuning methodology requires one training and one evaluation set. In order to keep continuity with our previous research, the training set T-3 was used in the training phase. Within the evaluation phase, a specialized (tuning) dataset should be used. The tuning set should be large enough and it should be affected by the target distortion of the “positive” samples. Further, both types of the “negative” samples, E and G (Section 2.1.3), should be equally represented in the set. Searching for the optimal setting on such datasets may guarantee finding a setting which would be appropriate to given requirements on the robust detector.

For this purpose, a tuning set on ten photos was created. These photos were captured under the condition specified in [7]. They were captured at six different locations. None of these photos were used while forming the training set. The tuning set consisted of labelled RGB object images of size 40 × 40 px. The labelled object images were created from the photos using an editor [19]. To create the dataset, 1000 unique “positive” and 4000 unique “negative” samples were gathered. The set was extended using artificial “positive” samples created by turning the images through the angle *φ*; that is, it consists of 4000 “positive” and 4000 “negative” samples.

#### 2.3.3. Datasets for Evaluation

In Section 2.1.3, the evaluation test sets, E and G, were mentioned. Each of these sets consisted of only 400 samples, which seems to be insufficient for a credible assessment of the meaning of the conversion. In order to get meaningful results, we formed new* expanded test sets*. These sets were created in the same spirit as the original test sets. Continuity of the marking was also maintained; that is, expanded test sets of the environment type were labelled as EX and expanded test sets of the grape type as GX. Two expanded test sets of each type were formed. In addition, a new type of test set was introduced. This set was labelled as* expanded standard test set* or SX. The set was not affected by the distortion.

All the expanded test sets comprised labelled RGB object images of the size 40 × 40 px. The labelled object images were created from vineyard row photos using the editor [19]. The sets EX and GX were based on a collection of ten unique vineyard row photos. Ten different photos were used for SX creation. The photos did not match with the photos used when creating the training and the tuning set. The photos were captured at six different locations under the conditions specified in [7].

An expanded test set, either EX or GX, consisted of 500 unique “positive” and 2000 unique “negative” samples. The sets were extended using artificial “positive” samples created by turning the images through the angle *φ*; that is, they consisted of 2000 “positive” and 2000 “negative” samples. The selection of the “positive” and of the “negative” samples followed the criteria stated in Section 2.1.3. Sets EX-*i* and GX-*i* with the same index *i* shared the same collection of “positive” samples. The standard set SX consisted of 2000 unique “positive” and 2000 unique “negative” samples.

#### 2.3.4. Inquire into the Sensitivity to the Rotation

The potential sensitivity of the detectors to the image rotation was the second issue to be investigated. Suspicion on the sensitivity came from the disproportion between the results acquired by the 10-fold CV or by the evaluation on the cut-outs and the results obtained by the evaluation on the test sets. The new expanded dataset, EX, GX, and SX, allowed us a detailed exploration of this issue. A comparison of evaluation results obtained on the expanded dataset with evaluation results obtained on the original datasets is the key. In order to get comparable results, the detectors S_1_, S_2_, and R tuned according to the proposed methodology, must be evaluated also on the sets E and G.

A high sensitivity of an assessed detector to the rotation would be noticeable from a comparison of evaluation results obtained on EX (GX) with evaluation results obtained on E (G). Similarity of these results would indicate the high sensitivity. Worse evaluation results obtained on EX (GX), rather than on E (G), would also confirm the high sensitivity. A low sensitivity of the detector would be visible from the comparison of evaluation results obtained on EX and GX with evaluation results obtained on SX. A worse performance on EX or GX, rather than on SX, would confirm the low sensitivity. All other results would signify its insensitivity to the rotation. In order to eliminate a potential influence of the proposed tuning methodology on the evaluation results, the new results obtained for E and G were compared with the original results.

## 3. Results and Discussion 

### 3.1. Optimal Settings of Detectors

Optimal settings of the detectors were determined using the presented methodology. In order to keep the continuity of our work, the performance measures (1a), (1b), and (1c) were used for the evaluation of the detector performance. It means that *M* = {accuracy, precision, recall}. The accuracy was used for the tuning of all former versions of the detectors. For this reason, the accuracy was chosen as the primary measure with the highest priority. Detection of all grapes in a photo is essential for applications such as yield estimation. Thus, the recall was taken as the secondary measure; and consequently, the precision was considered to be the tertiary one.

Depending on the version, the detectors have up to five tuning parameters. While the weighting coefficients *w* are bounded by the conditions *w*_R_, *w*_G_, *w*_B_ ∈ [0,1] and *w*_R_ + *w*_G_ + *w*_B_ = 1, the regularization constant *C*, likewise the kernel width *σ*, must be positive. Based on our previous experience, the following settings of the parameters were used within the search process: *X*_*C*_ = {1,10,100,1000}, *X*_*σ*_ = {1,10,20,30,40,100}, and *p* = 20. In such a way, sets Θ of all admissible settings *θ* were formed.

While the search for the optimal setting of S_1_ and S_2_ according to the proposed methodology does not require any intervention of human, the search for the optimal setting of R cannot be performed without the computer vision expert.

#### 3.1.1. Optimal Setting of Robust Detector with RBF Kernel

**Figure 4:**
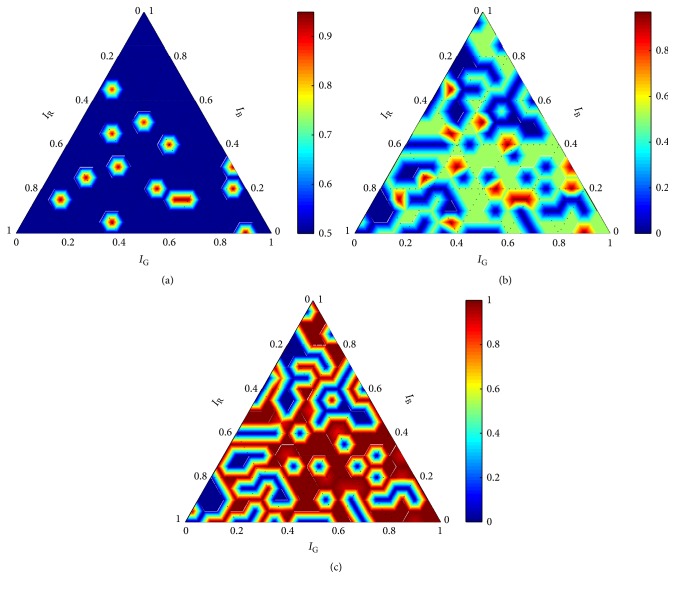
Performance of the robust detector with the RBF kernel for *C* = 10 and *σ* = 1 according to (a) accuracy, (b) precision, and (c) recall.

**Figure 5:**
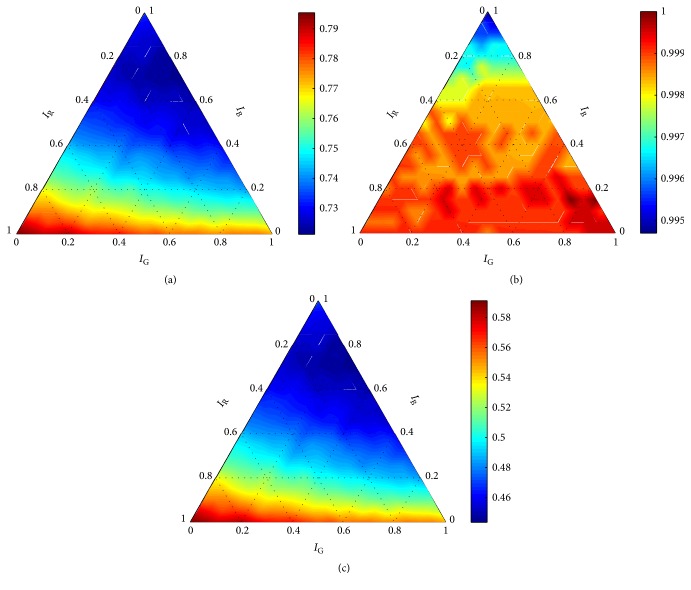
Performance of the robust detector with the RBF kernel for *C* = 10 and *σ* = 10 according to (a) accuracy, (b) precision, and (c) recall.

**Figure 6:**
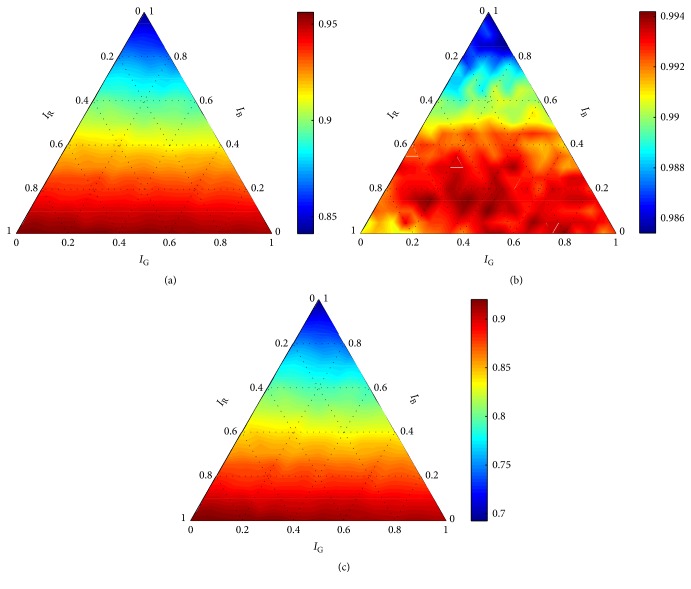
Performance of the robust detector with the RBF kernel for *C* = 10 and *σ* = 20 according to (a) accuracy, (b) precision, and (c) recall.

**Figure 7:**
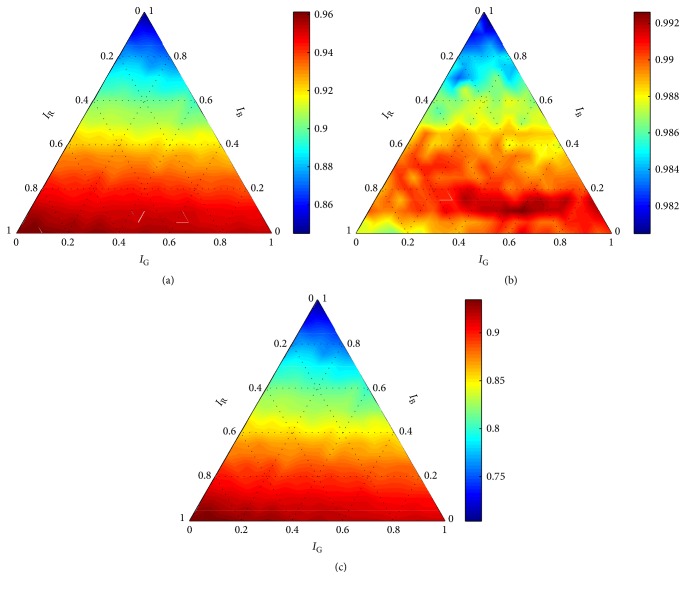
Performance of the robust detector with the RBF kernel for *C* = 10 and *σ* = 30 according to (a) accuracy, (b) precision, and (c) recall.

**Figure 8:**
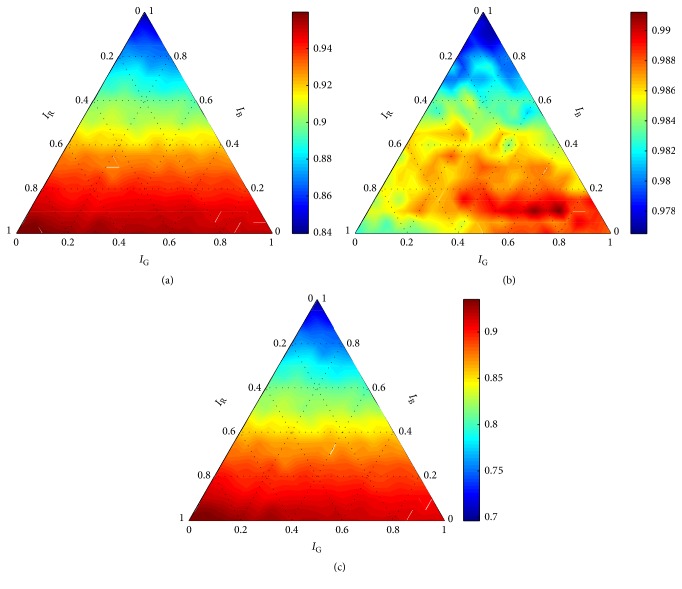
Performance of the robust detector with the RBF kernel for *C* = 10 and *σ* = 40 according to (a) accuracy, (b) precision, and (c) recall.

On the basis of data obtained within the training-evaluation process, 72 graphs were created. On the basis of their mutual comparison, we discovered that *σ* has much higher influence on the performance measured by accuracy and recall than *C*. Further, we found that both *C* and *σ* influence the performance only slightly from the perspective of precision. The main trends captured by the graphs can be demonstrated on graphs obtained for an arbitrary chosen setting of *C* and *σ* ∈ {1,10,20,30,40}. For this purpose, we chose results obtained for these values of *σ* and *C* = 10. The results are shown for all three measures in Figures 4–8.

The analysis of the 72 diagrams pointed out an abnormality in the results obtained for *σ* = 1 and ∀*C* ∈ *X*_*C*_. The abnormality is clearly visible in Figure 4, where the performance of the robust detector with the RBF kernel is shown for *C* = 10 and *σ* = 1. For example, accuracy (Figure 4(a)) is 0.5 for the majority of the settings **w** ∈ *W*; nevertheless, excellent scores were achieved for some of them. It is apparent that a very small change in the setting of the weighting coefficients would lead to a drastic drop in accuracy. The diagrams for the other measures (Figures 4(b) and 4(c)) show similar discrepancies. Very similar graphs were obtained for ∀*C* ∈ *X*_*C*_ in combination with *σ* = 1.

The seriousness of the discrepancies is even more apparent when comparing graphs obtained for *σ* = 1 with graphs obtained for higher values of *σ*. Graphs obtained for *C* = 10 and *σ* > 1 (Figures 5–8) show consistent trends for all performance measures. Such trends were obtained for ∀*C* ∈ *X*_*C*_ and ∀*σ* ∈ {*σ* ∈ *X*_*σ*_∣*σ* ≠ 1}. It is clear that *σ* = 1 cannot guarantee robustness of the final solution. Thus, all evaluation results, obtained for *σ* = 1, were eliminated from the further processing; that is, $\hat{\Theta }=\{(C,\sigma ,w)\in \Theta |\sigma \neq 1\}$. On the basis of formula (3), results summarized in Table 1 were obtained.

Diverse results were obtained for the measures *m* ∈ *M*. Although the measures have predefined priorities, meaningfulness of the results should be always considered before the globally optimal setting *θ*^*∗*^ is determined. It is apparent from the graphs (Figures 5–8) that the parameters *σ*, **w** had negligible influence on precision. When comparing all obtained graphs, we found that also *C* hardly influenced precision. Thus, precision was abandoned within the final assessment.

According to the remaining two measures, the setting providing the best performance was **w** = (0.95,0.05,0.00) and *C* = 10. However, the obtained results did not allow the direct determination of the optimal value of *σ*. Since accuracy had the higher priority, the optimal setting of *σ* was determined according to this measure. As is apparent in Figures 7 and 8, there was only insignificant difference in the performance measured using recall for *σ* = 30 and *σ* = 40. Thus, we selected *θ*^*∗*^ = (10,30, (0.95,0.05,0.00)) to be the globally optimal setting for the robust detector with the RBF kernel.

#### 3.1.2. Optimal Setting of Robust Detector with Linear Kernel

**Figure 9:**
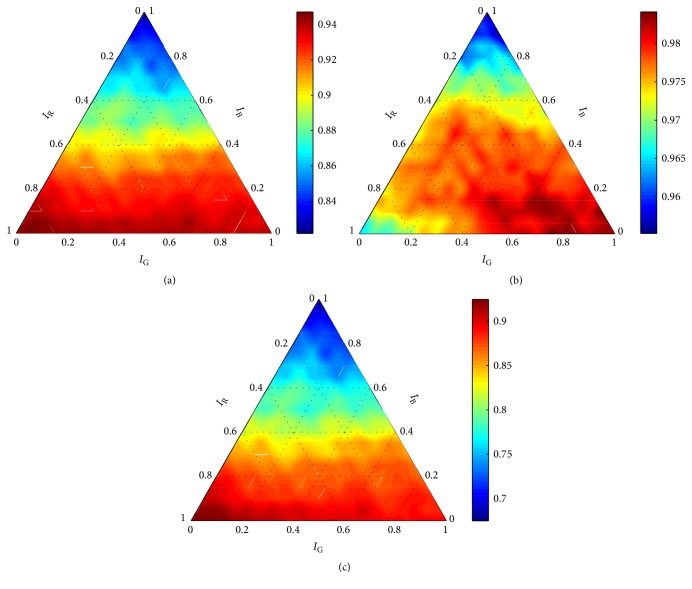
Performance of the robust detector with the linear kernel for *C* = 1 according to (a) accuracy, (b) precision, and (c) recall.

The analysis of the data obtained within the training-evaluation process was much simpler in the case of the robust detector with the linear kernel. At first, only 12 graphs were created on the basis of the obtained data. Secondly, the regularization constant *C* did not influence the performance of the detector; that is, identical graphs were obtained for ∀*C* ∈ *X*_*C*_. Thus, for the next explanation, we selected results obtained for *C* = 1 (Figure 9).

It is apparent in the graphs (Figure 9) that there was no anomaly in the evaluation results, and the detector reached high scores for all used measures. It means that $\hat{\Theta }=\Theta$. According to formula (3), settings summarized in Table 2 were determined to be optimal for ∀*m* ∈ *M*.

As is apparent in Figure 9, the weighting coefficients **w** had almost no influence on precision. Thus, just accuracy and recall were taken into account when the final decision was made. Since identical optimal settings were obtained for both measures, we can positively recommend the setting *θ*^*∗*^ = (1, (0.95,0.05,0.00)) as the globally optimal setting for the robust detector with the linear kernel. However, we should point out here that any *C* ∈ *X*_*C*_ might be used as well.

#### 3.1.3. Summary of Optimal Settings

Herein, we provide a summary of optimal settings for various versions of the detectors. The summary can be found in Table 3.

### 3.2. Evaluation of Detectors

In order to investigate the opened issues, evaluations of the detectors on the expanded as well as on the original datasets were done. The results obtained on the expanded sets, EX, GX and SX, are summarized in Table 4. The results obtained on the original sets, E and G, are stated in Tables 4 and 5, respectively.

### 3.3. Discussion of Evaluation Results

Two main issues were opened in this article. Our findings are presented in following text.

#### 3.3.1. Assessment of Conversion Importance

The importance of the conversion in the grape detectors was assessed on the basis of results obtained on EX, GX, and SX (Table 4). Let us consider the results obtained for the detectors with the linear kernel at first. From comparison of S_1_ and R with S_2_, it follows that the standard conversion according to the ITU-R recommendation, as well as the generalized conversion according to (2), enhanced scores obtained using accuracy and recall on EX and GX. The improvement is more evident for recall. Precision was slightly better for S_1_ for these datasets; however, a small downturn in precision was registered for R. Considering the significant improvement in recall, the conversions seemed to be valuable parts of the detectors. However, the results obtained on SX did not confirm these outcomes. Thus, using of a conversion for detectors with the linear kernel cannot be positively recommended.

In the case of the detectors with the RBF kernel, the results spoke definitely on behalf of the conversions. The detectors S_1_ and R outperformed the detector S_2_ in accuracy, and especially in recall, on all the expanded sets, that is, on EX, GX, and SX. Within all the experiments, the changes in precision were marginal. Let us focus now only on the detectors S_1_ and R. From the results, it is apparent that R always outperformed S_2_ in accuracy and recall. We considered the downturn of precision of R to be marginal. Indeed, it never fell below 0.9800. Thus, from the perspective of the performance, the robust detector with the RBF kernel can be positively recommended as the most reliable solution. With accuracy ≥ 94.37%, recall ≥ 0.9010, and precision ≥ 0.9816, the robust grape detector with the RBF kernel is fully comparable with the state-of-the-art solutions aimed at the detection of single grapes of white varieties.

#### 3.3.2. Inquire into the Sensitivity to the Rotation

As the first step, we compared the results obtained for the detectors S_1_ and S_2_ (Tables 5 and 6), tuned according to the proposed methodology, with the original results [9]. We found that the new results are almost identical to the original ones. We came to the conclusion that the tuning methodology did not influence the results significantly.

In the second step, we compared the results achieved by the detectors on type matching sets; that is, results obtained on E (G) were compared with results obtained on EX (GX). We found that all detectors tuned according to the proposed methodology achieved significantly better results on the expanded sets EX and GX (Table 4), rather than on the original sets E (Table 5) and G (Table 6). It is evident that the poor performance of the detectors, which was reported in [7, 9], was mainly due to the inappropriateness of the sets E and G. Thus, we recommended using exclusively the new expanded datasets for the evaluation.

In the third step, we compared the results achieved by the detectors on the expanded sets (Table 4). For both types of the kernels, we observed a connection between the performance of the detectors and the grayscale conversion. While the detectors S_2_ (without the conversion) had considerably lower recall on EX and GX than on SX, the robust detectors reached almost identical results on all these sets. The detectors S_1_ showed results on the borderline between S_2_ and R. Thus, we came to the conclusion that the detectors are sensitive to the image rotation; however, the sensitivity can be suppressed by the grayscale conversion. We further found that the robust detector with the RBF kernel is almost resistant to this distortion.

## 4. Conclusion

The grape detectors based on SVMs classifiers and HOG features were appropriate solutions for detection of single grapes of white varieties, supported by excellent results by the 10-fold CV and the evaluation on the real-life images. However, results obtained by the evaluation on the test sets prompted a thorough examination of the detector performance for a confirmation of its expected merits. Our results showed that the grayscale conversion should be excluded when the SVM classifier with the linear kernel is used in combination with the HOG features. Using the linear kernel and skipping the entire image preprocessing ensure a low time complexity of the final solution, while keeping excellent performance on standard datasets. Such solution might be used in applications where worse performance under unfavourable conditions is not critical, for example, in autonomous vineyard sprayers.

The robust grape detector with the RBF kernel is fully comparable with the state-of-the-art solutions aimed at detection of single grapes of white varieties. The detector has greater time complexity than a detector without the grayscale conversion, but this disadvantage is counterbalanced by its excellent performance. Since the robust grape detector with the RBF kernel provides excellent results under standard conditions, as well as under unfavourable conditions, we recommended its usage in applications where the high accuracy and recall are essential, for example, for the yield estimation.

In the presented application, the modification of the grayscale conversion proved to be valuable. We believe that its usage is not limited to the grape detection. The modification, together with the tool set for parameter optimization, as well as the novel visualization method introduced in this contribution, might be used in various application areas.

## Figures and Tables

**Table 1:** Optimal settings of the robust detector with the RBF kernel according to different measures. The results are ordered according to the priority of the measures.

	*C*	*σ*	**w**
*θ*_accuracy_^*∗*^	10	30	(0.95,0.05,0.00)
*θ*_recall_^*∗*^	10	40	(0.95,0.05,0.00)
*θ*_precision_^*∗*^	1	10	(0.00,0.85,0.15)

**Table 2:** Optimal settings of the robust detector with the linear kernel according to different measures. The results are ordered according to the priority of the measures.

	*C*	**w**
*θ*_accuracy_^*∗*^	1	(0.95,0.05,0.00)
*θ*_recall_^*∗*^	1	(0.95,0.05,0.00)
*θ*_precision_^*∗*^	1	(0.00,0.90,0.10)

**Table 3:** Optimal settings for various versions of the detectors where S_2_ is detector without any conversion, S_1_ is detector with the conversion according to ITU-R recommendation BT.601, and R is detector with optimally set-up generalized conversion (2).

Version	Linear kernel		RBF kernel		
	*C*	**w**	*C*	*σ*	**w**
S_2_	1	—	1	40	—
S_1_	1	—	10	30	—
R	1	(0.95,0.05,0.00)	10	30	(0.95,0.05,0.00)

**Table 4:** Evaluation of the detectors trained on T-3, set-up according to Table 3, on new datasets of environment type (EX), grape type (GX), and standard type (SX), where |*P* | = 2000 and |*N* | = 2000. Following versions of the detectors evaluated using the measures (1a), (1b), and (1c): S_2_ is detector without any conversion, S_1_ is detector with the conversion according to ITU-R recommendation BT.601, and R is detector with the generalized conversion (2).

Kernel	Version	EX-1			EX-2			GX-1			GX-2			SX		
		Accuracy	Precision	Recall	Accuracy	Precision	Recall	Accuracy	Precision	Recall	Accuracy	Precision	Recall	Accuracy	Precision	Recall
Linear	S_2_	91.73%	0.9755	0.8560	90.93%	0.9712	0.8435	91.87%	0.9788	0.8560	91.10%	0.9751	0.8435	95.23%	0.9804	0.9230
	S_1_	94.15%	0.9768	0.9045	92.07%	0.9714	0.8670	94.63%	0.9869	0.9045	92.40%	0.9786	0.8670	94.73%	0.9833	0.9100
	R	94.20%	0.9580	0.9245	93.07%	0.9600	0.8990	95.07%	0.9757	0.9245	93.83%	0.9756	0.8990	94.70%	0.9671	0.9255

RBF	S_2_	93.27%	0.9909	0.8735	92.20%	0.9879	0.8545	93.25%	0.9904	0.8735	92.20%	0.9879	0.8545	95.83%	0.9909	0.9250
	S_1_	94.87%	0.9854	0.9110	93.73%	0.9888	0.8845	95.33%	0.9940	0.9120	93.63%	0.9866	0.8845	95.85%	0.9914	0.9250
	R	95.85%	0.9816	0.9345	94.37%	0.9852	0.9010	96.35%	0.9920	0.9345	94.50%	0.9879	0.9010	95.87%	0.9873	0.9295

**Table 5:** Evaluation of the detectors trained on T-3, set-up according to Table 3, on original datasets of environment type (E), where |*P* | = 200 and |*N* | = 200. Following versions of the detectors evaluated using the measures (1a), (1b), and (1c): S_2_ is detector without any conversion, S_1_ is detector with the conversion according to ITU-R recommendation BT.601, and R is detector with the generalized conversion (2).

Kernel	Version	E-1			E-2			E-3			E-4			E-5		
		Accuracy	Precision	Recall	Accuracy	Precision	Recall	Accuracy	Precision	Recall	Accuracy	Precision	Recall	Accuracy	Precision	Recall
Linear	S_2_	86.50%	1.0000	0.7300	87.25%	0.9869	0.7550	85.50%	0.9671	0.7350	86.25%	0.9801	0.7400	84.50%	0.9859	0.7000
	S_1_	88.50%	0.9873	0.7800	88.50%	0.9753	0.7900	87.50%	0.9573	0.7850	87.75%	0.9748	0.7750	87.00%	0.9868	0.7500
	R	88.25%	0.9693	0.7900	90.25%	0.9763	0.8250	88.75%	0.9586	0.8100	88.25%	0.9693	0.7900	87.75%	0.9809	0.7700

RBF	S_2_	88.00%	0.9935	0.7650	89.50%	0.9938	0.7950	88.00%	0.9872	0.7700	87.75%	0.9935	0.7600	86.25%	1.0000	0.7250
	S_1_	89.50%	1.0000	0.7900	90.25%	0.9879	0.8150	89.00%	0.9815	0.7950	89.25%	0.9937	0.7900	87.50%	1.0000	0.7500
	R	88.50%	0.9936	0.7750	89.50%	0.9877	0.8000	88.50%	0.9812	0.7850	88.00%	0.9634	0.7900	86.75%	0.9933	0.7400

**Table 6:** Evaluation of the detectors trained on T-3, set-up according to Table 3, on original datasets of grape type (G), where |*P* | = 200 and |*N* | = 200. Following versions of the detectors evaluated using the measures (1a), (1b), and (1c): S_2_ is detector without any conversion, S_1_ is detector with the conversion according to ITU-R recommendation BT.601, and R is detector with the generalized conversion (2).

Kernel	Version	G-1			G-2			G-3			G-4			G-5		
		Accuracy	Precision	Recall	Accuracy	Precision	Recall	Accuracy	Precision	Recall	Accuracy	Precision	Recall	Accuracy	Precision	Recall
Linear	S_2_	86.25%	0.9560	0.7600	81.00%	0.9697	0.6400	87.00%	0.9933	0.7450	85.75%	0.9673	0.7400	84.75%	0.9728	0.7150
	S_1_	86.25%	0.9618	0.7550	79.25%	0.9606	0.6100	86.00%	0.9865	0.7300	87.50%	0.9870	0.7600	84.75%	0.9793	0.7100
	R	87.75%	0.9576	0.7900	80.25%	0.9618	0.6300	86.50%	0.9803	0.7450	86.75%	0.9565	0.7700	86.00%	0.9500	0.7600

RBF	S_2_	88.75%	0.9874	0.7850	81.00%	0.9844	0.6300	89.00%	1.0000	0.7800	87.25%	0.9934	0.7500	88.00%	0.9935	0.7650
	S_1_	89.50%	0.9877	0.8000	81.00%	0.9844	0.6300	88.25%	1.0000	0.7650	87.25%	0.9869	0.7550	87.75%	0.9935	0.7600
	R	89.00%	0.9699	0.8050	82.25%	0.9924	0.6500	87.00%	0.9933	0.7450	87.25%	0.9806	0.7600	87.75%	0.9935	0.7600
